# Magnitude and risk factors of abortion among regular female students in Wolaita Sodo University, Ethiopia

**DOI:** 10.1186/1472-6874-14-50

**Published:** 2014-03-26

**Authors:** Amha Admasie Gelaye, Kalemelekot Nigussie Taye, Tesfa Mekonen

**Affiliations:** 1 School of Public Health, College of Health Sciences and Medicine, Wolaita Sodo University, Wolaita Sodo, Ethiopia; 2 School of Nursing and Midwifery, College of Health Sciences and Medicine, Wolaita Sodo University, Wolaita Sodo, Ethiopia

**Keywords:** Abortion, Induced abortion, Abortion law, University students, Youth sexual reproductive health, Youth sexual experience

## Abstract

**Background:**

Induced abortion is one of the greatest human rights dilemmas of our time. Yet, abortion is a very common experience in every culture and society. According to the World Health Organization, Ethiopia had the fifth largest number of maternal deaths in 2005 and unsafe abortion was estimated to account for 32% of all maternal deaths in Ethiopia. Youth are disproportionately affected by the consequences of unsafe abortion. The objective of this study was, therefore, to determine the magnitude and identify factors associated with abortion among female Wolaita Sodo University students.

**Methods:**

A descriptive, cross-sectional study was conducted in Wolaita Sodo University between May and June 2011. Data were collected from 493 randomly selected female students using structured and pre-tested questionnaires.

**Results:**

The rate of abortion among students was found to be 65 per 1000 women, making it three fold the national rate of abortion for Ethiopia (23/1000 women aged 15–44). Virtually all of the abortions (96.9%) were induced and only half (16) were reported to be safe. Students with history of alcohol use, who are first-year and those enrolled in faculties with no post-Grade 10 Natural Science background had higher risk of abortion than their counterparts. About 23.7% reported sexual experience. Less than half of the respondents (44%) ever heard of emergency contraception and only 35.9% of those who are sexually experienced ever used condom.

**Conclusions:**

High rate of abortion was detected among female Wolaita Sodo University students and half of the abortions took place/initiated under unsafe circumstances. Knowledge of students on legal and safe abortion services was found to be considerably poor. It is imperative that improved sexual health education, with focus on safe and legal abortion services is rendered and wider availability of Youth Friendly family planning services are realized in Universities and other places where young men and women congregate.

## Background

Every day, approximately 1000 women die from preventable causes related to pregnancy and childbirth and 99% of all maternal deaths occur in developing countries [1]. Evidence also suggests that adolescents face a higher risk of complications and death as a result of pregnancy than older women [1,2].

According to the World Health Organization, Ethiopia had the fifth largest number of maternal deaths in 2005 [3]. The maternal mortality rate (MMR) in Ethiopia was estimated at 673 deaths per 100 000 live births in the year 2005, and unsafe abortion and its complications were estimated to account for 32% of all maternal deaths [4-6].

Abortion is a very common experience in every culture and society [7,8]. Out of the 210 million pregnancies that occur each year globally, an estimated 46 million (22 per cent) end up in induced abortion, in relation to that, 19 million women experience unsafe abortions annually [7-10]. In the Eastern African region, 2.3 million abortions (virtually all unsafe, < 0.05 safe) occurred in 2003, making the abortion rate in the region 39 per 1000 women in reproductive age [7]. Similarly, some localized and national studies conducted in Ethiopia, document that the prevalence of induced abortion and its negative consequences are increasing from time to time in the country [11-14].

Review of risk factors of abortion shows that several variables have been implicated as risk factors of abortion in studies conducted across different countries. In U.S. poor women were reported to experience abortion more often than their counter parts [10]. Elsewhere, a study conducted in Hungary, concluded that reliable contraceptive methods were used significantly less frequently by the aborters than by the control group and the likelihood of abortion was significantly lower among those informed by a health-care provider [15]. A study conducted locally in Northern Ethiopia stated that, place of residence, marital status, contraceptive use, number of pregnancies and level of education attained by the women were found to be significantly and independently associated with induced abortion; while, fear of family criticism was mentioned as reason for resorting to abortion by Jimma University students [14]. Two other studies documented sexual violence or rape to be primary cause for seeking abortion 20-25% of the cases [16].

Recent changes in abortion law in Ethiopia are believed to pave the way for access to safe abortion services and subsequently reduce the burden of unsafe abortion and its complications and maternal death [5]. Notwithstanding the new law, however, almost six in 10 abortions in Ethiopia are unsafe [12].

Segmented evidence on the problem of abortion, its risk factors, the preference of care seeking behaviour and knowledge of legal status of abortion is imperative to guiding informed reproductive health interventions to youth in the university as well as similar facilities and possibly to shaping national and international policy and strategy to address maternal mortality and morbidity. Data on the problem of abortion among University students is particularly scanty [13]. It was therefore, necessary and timely to attempt to contribute to this end; this research tried to do just that.

## Methods

### Study design

The study employed a cross sectional study design to assess the magnitude of abortion among 514 female students in Wolaita Sodo University in 2010/11 academic year. Post-hoc control group was utilized to explore factors associated with abortion.

### Description of the study area

Wolaita Sodo University is a government University located in Wolaita Sodo town, Southern Ethiopia. The total students enrolled in on a regular basis, during the study period, was 8592. Out of these, 1302 were female students.

### Study population and sample

Participants were selected out of all female students enrolled in regular program in Wolaita Sodo University during the 2010/11 academic period. A stratified cluster sampling technique was employed to proportionally recruit students from all schools and faculties of the University.

The required sample size for the study was determined by using the single population proportion formula: n = Z^2^pq/d^2^. A 20% rate of abortion was taken from a community based study in north Ethiopia to estimate the sample size, with a margin of error 5%, 95% confidence level and non-response rate of 5%. design effect of 2 was used to account for the cluster sampling involved. The final sample size was determined to be 514.

### Data collection and analysis

Self administered questionnaires were used for data collection. Data collected were entered into and cleared using Epi-INFO software version 3.5.1 and then transported to SPSS version 16 for further statistical analysis. Bi-variate regression analysis was used to look for association between predictors and dependent variables [Age, Academic year, Economic Status, Faculty, Religion, Home residence, Condom Use and Emergency Contraceptive use] and dependent variables [Sexual practice and Abortion] Multivariate logistic regression analysis was done to control for confounding and identify the most important determinant variables. Odds ratio and the respective 95% confidence intervals were used to assess the statistical significance of association among the variables.

### Data quality assurance

A pre-test of the data collection tool was carried out in Arba-Minch University in a similar setting and adjustments were made accordingly. Questionnaires were prepared in English (Instructional media of the University) as well as in Amharic. Students were explained the purpose of the study and were assured of confidentiality and the need for providing honest answers. In addition to specific instructions on the questionnaire, participants were given clear oral guidelines on filling out the questionnaire.

### Ethical issues

The study was approved by the Research and Publication committee of the Wolaita Sodo University. Written consent was obtained from Research and Publication committee of the University. Verbal informed consent was obtained from the study participants. Necessary precaution was made to ensure confidentiality. Students were thoroughly explained on their rights and the purpose of the research.

## Results

### Abortion and associated factors

At the beginning of the study 530 female students were identified out of which 514 consented and 493 provided questionnaire responses yielding a response rate of 93%. A total of 32 abortions were reported in the court, making the rate of abortion among Wolaita Sodo University 65 per 1000 women (n = 32/493. Out of these 9.4% were recurrent abortions.

The age of the study participants ranged from 17 to 29 with mean ± SD of 20.0 ± 1.3 years. First year students constituted the major proportion (58.4%) followed by second year students (22.7%). About half (50.9%) of the respondents were Orthodox Christians fallowed by Protestants and Muslims which made up 29% (143) and 14.8% (73) respectively. Virtually all of the students were single 484 (98%). The reported monthly earnings (pocket money) of the students ranged from 0 to 1500ETB with mean ± SD 279 ± 208ETB (mean roughly 20 USD). Most 145 (29.4%) of the respondents were enrolled under the faculty of social sciences and humanities, followed by students in Natural and Computational Sciences faculty and Agriculture faculty, which accounted 19.1% and 16.8% respectively. Table 1 presents detailed socio demographic characteristics of the students.

**Table 1:** Background characteristics of study population/female students in Wolaita Sodo University, June 2011

**Characteristics**	**Responses**	**Frequency**	**Percentage**
**Age (n = 489)**	17 – 19	169	34.6
	20 – 24	314	64.2
	25 +	6	1.2
**Class year (n = 492)**	1st year	288	58.5
	2nd year	112	22.8
	3rd year	83	16.9
	4th year	9	1.8
**Religion (n = 493)**	Orthodox Christian	251	50.9
	Protestants	143	29
	Muslim	73	14.8
	Catholic	20	4.1
	Others	6	1.2
**Marital status**	Single	484	98
	Married	9	2
**Permanent residence (n = 493)**	Urban	203	41.2
	Semi-Urban	180	36.5
	Rural	110	22.3
**Faculty/school (n = 491)**	Agriculture	83	16.9
	Health Sciences	31	6.3
	Natural & Computational Sciences	94	19.1
	Social Sciences & Humanities	145	29.5
	Business and Economics	65	13.2
	Law	22	4.5
	Engineering	51	10.4
**Pocket money/months (n = 493)**	1st quartile (≤ 183 birr)	110	22.3
	2nd quartile (183–233)	148	30
	3rd quartile (234–319)	120	24.3
	4th quartile (≥320)	115	23.3

The percentage of pregnancy experienced in the last twelve months was 7.7% (34) and in 29 (85.3%) of the cases, pregnancy was unwanted. In 37.3% education was considered the primary reason for not wanting pregnancy; in 4 (12.5%) pregnancy was unwanted because it occurred due to low risk perception of pregnancy; in 3 (9.4%) due to contraceptive failure, in 3 (9.4%) others due to rape, in 2 (6.3%) due to inappropriate use of contraceptive and in 1 because it was an incest pregnancy.

Virtually all abortions (96.9%) were induced abortions out of which 34% were self-induced. Only half (16) were reported to be safe, that is, they were performed by a trained health professional in standard health institution. Most(11) of those who had initiated the induction of abortion by themselves reported used excessive drugs (like Ampicillin) and some (8) used traditional remedies like “Embway” - a traditional herbal medicine (a variety of nightshade, Official name: Solanum marginatum L. f) [17] and the remaining (7) reported use of some kind of physical means to initiate abortion by themselves.

**Figure 1:**
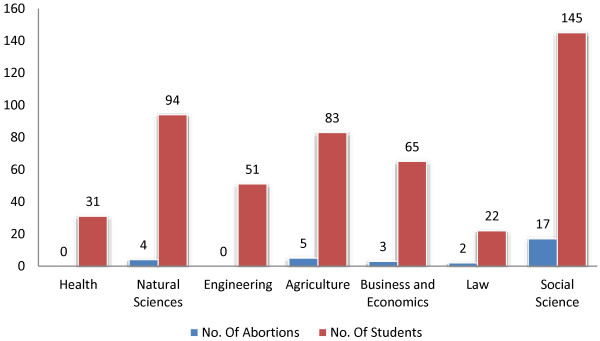
Number of abortions with respective number of students in each faculty/school, Wolaita Sodo University, June 2011 (n=492).

The rate of abortion varied in students from different schools and faculties of the University from as low as 0% among students in the Faculty of Health to as high as 11.7 among the Faculty of Social Sciences students (Figure 1). In a similar manner, students in faculties with no post-Grade 10 Science background were about three times more likely to have experienced abortion than students with post-Grade-10 natural science background [adjusted odds ratio 2.91(1.31-6.46) (Figure 1).

The study also identified that use of alcohol and class year of students had independent and statistically significant association with likelihood of experience of abortion. Students who reported use of alcohol had about four times more risk of experiencing abortion than students who never used alcohol [adjusted odds ratio 3.95(1.63-11.11)]. Additionally, the odds of having had abortion were again four times as high among fist year students as compared to second year and higher class year students [adjusted odds ratio 3.98(1.50-10.53). On the other hand, reported ever use of condom and emergency contraception did not appear to be protective against abortion [odds ratio 0.47(0.18-1.25) and 0.92(0.38-2.28)] respectively. Table 2 depicts the multivariate regression analysis results for factors associated with abortion.

**Table 2:** Factors affecting practice/experience of abortion among students in Wolaita Sodo University, June 2011 (n = 493)

**Characteristics**	**Ever had abortion**		**Crude OR**	**Adjusted OR**
	**Yes (n = 32)**	**No (n = 461)**		
**Age in years**				
≤ 20	19	303	1.00	1.00
> 20	13	158	1.31 (0.63-2.73)	1.47 (0.63-3.29)
**Religion**				
Orthodox	12	240	1.18 (0.23-4.31)	1.08 (0.28-4.18)
Protestant	15	129	2.75 (0.77-9.80)	3.13 (0.85-11.63)
Catholic	1	20	1.18 (0.11-12.04)	1.40 (0.13-14.92)
Muslim	3	71	1.00	1.00
**Class year**				
1st Year	27	261	4.13 (1.57-10.99)	3.98 (1.50-10.53)*
2nd or Above	5	200	1.00	1.00
**Monthly pocket money [ETB]**				
≤280	22	258	1.00	1.00
> 280	10	203	1.73 (0.80-3.73)	1.24 (0.40-3.82
**Faculty**				
Social, FBE & Law	22	210	2.91 (1.31-6.46)	2.48 (1.09-5.66)*
Natural and Health	9	250	1.00	1.00
**Home residence**				
Urban	10	193	1.00	1.00
Semi-Urban	13	167	1.72 (0.68-4.37)	1.37 (0.48-4.03)
Rural	9	101	1.15 (0.47-2.77)	1.04 (.04-2.62)
**Alcohol use**				
Yes	9	45	3.53 (1.54-8.06)	3.95 (1.63-11.11)*
No	23	416	1.00	1.00
**Ever used condom**				
Yes	7	35	1.00	1.00
NO	19	45	1.10 (0.44-2.65)	1.07 (0.30-3.61)
**Ever Used EC**				
Yes	11	34	1.00	1.00
NO	15	43	2.11 (0.80-5.58)	1.05 (0.31-3.52)

*Statistically Significant associations, Adjusted odds ratios.

**Figure 2:**
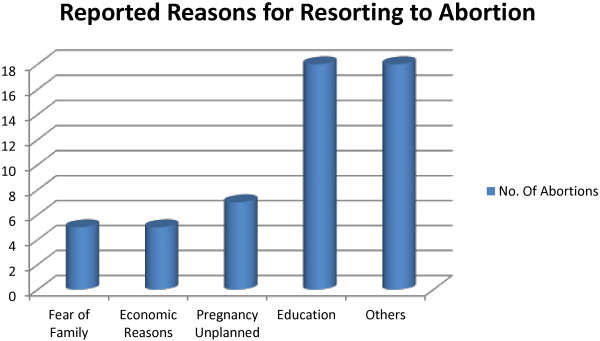
Reported causes for resorting towards abortion among Wolaita Sodo University Students, June 2011 (n=32), more than one factor may be reported.

Several conditions were implicated by study participants as underlying causes for opting to undergo abortion. Some are illustrated in the Figure 2.

Study participants reported several places where abortion was performed/initiated. In this regard, the leading spot where most abortions were performed/initiated was traditional abortionist’s home, where 9(28%) of the abortions were induced. Government health institutions follow, accounting 25% followed by private health institutions and NGO clinics, which jointly served for another 25% of the reported abortions. The remaining (22%) abortions were reportedly initiated either at own home or at a relative’s home.

Three fourths of those who undergone abortion reported to have experienced at least one complication following abortion. The most frequently experienced complication being excessive bleeding, which occurred in 21.9% of the cases. Tears (vaginal, uterine, cervical, etc.) were reported in 18.8%, severe pain in 15.6%, infection in 12.5% and retained product of conceptus (incomplete abortion) were reported in 6.3% of the cases.

Respondents also identified several different individuals or institutions as source of information for abortion services. Female peers served as main source of information in 28.1% of the cases while health professionals and partners in 25% each, and male peer in 18.8% of the cases. On the other hand, family and school served as source of information for abortion services in only 6.3% and 3% of the cases respectively.

### Sexual experience, condom use and emergency contraception

Close to a quarter of respondents (23.7%) were sexually active. Age at first sex ranged from as early as 12 years to 23 years with mean ± SD 18.75 ± 1.5 years. Most (81.8%) of those who are sexually experienced did so between the ages 18 and 20 and only 39 (33.3%) of them reported use of condom at first sex.

Sexual experience appeared to be inversely related with class year, with the highest proportion being 27.3 percent among first year students as compared to 20.5% and 16.3% among second and third year and above students respectively. Accordingly 1st year students were found to be 2.5 times more likely to have had sex than third year or above students (95% CI 1.2 – 5.4). Similarly, students who are 20 or younger had significantly higher sexual exposure than those who are older than 20. [OR = 1.8, (95% CI 1.1 – 2.9)]. Alcohol use was also found to have statistically significant association with sexual exposure, with those students who ever had alcohol having 5.5 times more likelihood of having sex than their counterparts (95% CI = 1.9 - 16).

Meanwhile, only 35.9% of those who are sexually experienced have ever used condom and 47.9% the same group admitted that they had had at least one unprotected sex (sex without condom) over the last twelve months. Condom use appeared to be higher for students with urban residence 75.5% followed by students from semi-urban areas (34.2%). Students who came from rural areas had the least condom usage (20%).

Less than half of the respondents (44%) ever heard of emergency contraception. Knowledge on emergency contraception increased with increase in class year, accordingly, it was 38.8%, 50% and 53.3% for first year, second year and third and above students respectively. Only 47 (40.2%) of the sexually experienced respondents ever used emergency contraception, with only 45% reporting use of EC within the first 24 hours of unprotected sex and 31.2% reported use.

### Knowledge on legal issues of abortion

When it comes to knowledge on Ethiopian abortion law there was sort of bewilderment among participants. Of those who have given responses regarding legal condition of abortion in Ethiopia, 172 (38.8%) replied that Ethiopian law allows abortion while 157 (35.5%) replied it did not and the remaining 114 (25.7%) answered that they didn’t know whether or not abortion is legally allowed in Ethiopia.

From the 172 (38.8%) respondents who claimed abortion is legally allowed, 65 (37.8%) identified serious mental and physical problems as ground for legal abortion; 77 (44.8%) identified pregnancy resulting from rape; 62 (36.3%) identified incest pregnancy; 45 (26.2%) identified minor pregnancies and 72 (41.7%) foetal abnormalities. Significant proportion (25 and 18%) claimed that abortion is allowed for all unwanted pregnancies and for all economic reasons respectively. However, out of all study participants, only 24(4.9%) properly identified all the conditions provided, under which abortion is legally allowed in Ethiopia. On the other hand, 34 (6.9%) identified at least three proper conditions; 39 (7.9%) at least two and 101 (22.8%) identified one proper condition as a basis for legal abortion.

Participants were asked to state conditions/grounds under which they thought abortion should be legalized. Accordingly 62 (12.6%) responded abortion should be available on demand while 255 (51.7%) rejected the idea of providing abortion services on demand. Asked to provide their description of the Ethiopian abortion law, more than half of the respondents (50.7%) did not provide descriptions or simply replied “don’t know”, while smaller proportions, 17.6% and 16% provided opposing views; describing it ‘restrictive’ and ‘legalized’ respectively. Another 10% described it to be appropriate. Similarly, respondents were asked their opinion as to whether more legalization of abortion law was better for maternal health. More people (26%) agreed that more legalization was better for maternal health than opposed (20%). Table 3 depicts the complete list of conditions with their respective percentages.

**Table 3:** Attitude of Wolaita Sodo University students with regard to conditions for legalization of abortion in Ethiopia, June 2011 (n = 493)

**Conditions for which abortion should be legalized**	**Favour # (%)**	**Oppose # (%)**
On demand	62 (12.6%)	255 (51.7%)
Economic reasons	184 (37.3%)	167 (33.9%)
Health problems	173 (35.1%)	176 (35.7%)
In case of rape	90 (18.3%)	235 (47.7%)
For spacing	62 (12.6%)	260 (52.7%)
For students	36 (7.3%)	274 (55.6%)
For unwanted pregnancy	31 (6.3%)	277 (56.2%)

Students were asked if they were to use abortion services for unwanted pregnancies, provided that abortion services are legalized. Only 18.9% of the students responded that they would resort to abortion for unwanted pregnancy. Most of them (57.4%) replied they wouldn’t opt to abortion even if it were to be legalized. Several factors were raised as primary reasons for declining abortion; the most important one being the belief that ‘abortion was against God’s will’ cited by 214 (43.4%) respondents. The other important reasons provided was a moral issue, that is, considering abortion as murder, as reported by 174 (35.3%) study participants.

### Barriers to safe abortion service utilization

Out of the total of 32 individuals who reported to have undergone abortion, appointments were perceived to be barriers/difficulties for service use in 6 (18.8%), non cooperative staff in 4 (12.5%), payment before service in 3 (9.4%), buying drugs and other supplies before service in 3 (9.4%), and absence of service provider was reported in 3 (9.4%). Participants reported to have incurred varying service costs ranging from ETB 40 to 1500 with median cost ETB 250 with IQR ETB 570.

## Discussion

This study revealed very worrisome figures of unsafe abortion and unsafe sexual practices among female Wolaita Sodo University students. The rate of abortion among WSU students was found to be 65 per 1000 women and virtually all of the abortions (96.9%) were induced and only half or 16 were reported to be safe. Study participants reported using a range of traditional and physical methods for inducing abortion. This rate of abortion amounts threefold the national rate of abortion for Ethiopia (23/1000 women aged 15–44); more than threefold the rate of abortion among first year medical students in Mexico City (2%) and significantly higher than the rate of abortion in Jima university which was accounted (4%) [13].

The findings of this study strongly imply that despite the recent policy changes to liberalize abortion in an effort to reduce unsafe abortion and subsequent maternal mortality, unsafe abortion remains to be a major problem affecting significant number of youth women.

The higher rate of abortion among female Wolaita Sodo University students may partly be explained by type of the study subjects. According to studies, youth carry significantly high proportion of the burden of abortion in their communities [18,19]. Regardless of the type of the study population, however, the rate of abortion among Wolaita Sodo University students can be considered very high.

Virtually all of the abortion in this study was induced accounting 96% of abortions. This figure is much higher than the rate of induced abortion reported by community based studies conducted in North west Ethiopia, Harar, and Eritrea, where only 4.8%, 14.4% and 11% of the abortion were reported to be induced. [11,20,21].

The high rate of abortion among Wolaita Sodo University students where sexual exposure appeared relatively low, is particularly troubling and may indicate that most sexual intercourses are unprotected and unsafe and hence raise serious concerns with risk of HIV/AIDS and other STDs.

With regard to risk factors, results of logistic regression analysis of the current study revealed that, alcohol use had statistically significant association with experience of abortion. Students who ever had alcohol were found to be up to four times more likely to have experienced abortion than students who never had alcohol. This is consistent with current evidence on the relationship between alcohol and risky sexual behaviour. Several studies concluded that alcohol was strongly associated with decreased protective behaviours among younger individuals [22,23]. Similarly, students from faculties and departments with high school level and beyond science education [Faculty of Health, Natural Sciences, Agriculture and Engineering] had a significantly lower risk of abortion as compared to students faculties with no post grade 10 level science course, [Faculty of Social Sciences, Faculty of Business and Economics and School of Law] (Figure 1). This might be related to a possible preventive role offered by a relatively better knowledge of sexuality and reproductive health gained as part of their natural sciences and health science courses. The multivariate logistic regression also revealed that first year students had a significantly higher risk of experiencing abortion than second year or above students. This is in agreement with results of logistic regression on sexual experience, where, in a similar fashion, the same group of students were found to be more likely to be sexually experienced. This further strengthens other findings that significant number of students are engaged in unprotected sex and hence prone to unintended pregnancy, abortion and STIs.

On the other hand, religious affiliation, age of students and monthly pocket money did not show significant association with the likelihood of having abortion. Similarly, ever use of emergency contraception and condom, did not appear to be protective against abortion in this study. Inconsistent use of condom as well as emergency contraception could explain this paradox. As presented earlier, significant number of students admitted practicing unprotected sex at least in one occasion over the last 12 months. In agreement with this, only 18% of respondents who reported knowing about emergency contraception knew the correct time frame in which emergency contraceptives must be used to be effective. Further research on knowledge and practice of students on emergency contraception in particular and contraceptive methods in general may prove helpful to explore possible barriers to contraceptive use and may unveil areas of action to enhance the reproductive health of students.

Several issues were mentioned by study participants as most important reasons for resorting to abortion. These reasons appear to underscore their understanding of the responsibilities of parenthood and family life. Three-fourths of women cited concern for or responsibility to other individuals; three-fourths said they couldn't afford a child; three-fourths said that having a baby would interfere with work, school or the ability to care for dependents; and half said they did not want to be a single parent or were having problems with their husband or partner [23].

As part of law reforms in Ethiopia in 2005 the penal code was revised to broaden the indications under which abortion is permitted. Termination of pregnancy is now legal when the pregnancy results from rape or incest, when continuation of pregnancy dangers the health or life of the women or the foetus, in case of foetal impairment, for women with physical or mental disabilities [4,11]. Despite the relative liberalization and despite the fact that several institutions in the town provided safe abortion services, the fact that significant proportion of students resorted to traditional and unsafe services indicates that access to safe abortion remains to be a problem. Furthermore, out of all study participants, only 24(4.9%) properly identified all the conditions under which abortion is legally allowed in Ethiopia. This demonstrates that liberalization of abortion by itself is not enough and that, in order to ensure that legislative changes improve reproductive health, women must know the legal options they have in the case of unwanted pregnancy. This is in agreement with findings of study conducted in South Africa, where abortion is legal, yet unmet need for abortion information resulted in significant occurrence of unsafe and illegal abortion [19,24].

Study participants raised several issues as barriers to accessing safe abortion services. In this regard, participants mentioned appointments, non cooperative staff and cost. Some study participants spent as much as 1500 ETB (almost 100 US dollars). Such a cost might serve as a huge barrier to this particular segment of the population with relatively limited financial means and deter them from accessing safe abortion services.

## Conclusion

In concussion, the rate of abortion among Wolaita Sodo University Students was higher as compared to most local and other rates elsewhere. It amounted three times as high as the rate for the general population in Ethiopia. Even higher rates of abortion might be detected by use of more robust methods. Moreover, alarmingly higher proportions of abortions (50%) were performed or initiated under unsafe circumstances and three fourths of those who had abortion suffered one or more complications. Students who ever used alcohol, who were in their first year and those without natural science backgrounds had significantly higher risk of abortion as compared to their counter parts. Knowledge of students on legal issues of abortion was very low; very few students properly stated all the conditions for legal abortion in Ethiopia. Risky sexual behaviours were widespread and knowledge and practice of students on healthy reproductive health behaviour, including emergency contraception and condom use were found to be very low.

### Recommendations

It is imperative that improved sexual health education is rendered and wider availability of Youth Friendly family planning services are realized in Universities and other places where young men and women congregate. Institutions providing safe abortion services, should devise strategies to reach out for youth who are in need of their services and prevent youth from resorting to unsafe abortionists and hence the grave complications of unsafe abortion. Wolaita Sodo University clinic should devise a way to make contraceptives, especially emergency contraceptives available for those in need, overcoming privacy barriers. Information, Education and Communications (IEC) programs on youth reproductive health should be properly tailored to address topics on unwanted pregnancy and safe abortion, especially to fill the knowledge gap of students with regard to legal issues surrounding abortion (Ethiopian abortion law) and safe abortion services. Expanding access to emergency contraception and condom distribution with focus on drinking establishment which students often use, might be equally important. Finally, alongside other efforts, lobbying for further liberalization of abortion services may serve to overcome perceived unnecessary barriers to access to safe abortion services by youth students.

### Strength and limitations of the study

#### Strength

The study was the first of its kind in the study population, accordingly provided important evidence on pressing reproductive health issues among university students. Data were collected at the end of academic calendar; hence the possibility of obtaining full year information about students was enhanced. The study involved a reasonably large sample size, leading to a fairly representative figure.

#### Limitations

Owing to the sensitive nature of the subject investigated and the fact that the research mainly depended on data from respondents, there could have been a room for social desirability bias with possible underestimation of the true prevalence of abortion among the students. The small number abortion cases accrued might undermine the power of statistical tests.

## Competing interest

The authors declare that they have no competing interests.

## Authors’ contribution

AAG conceived the study. AAG and KNT equally participated in the design, data collection, statistical analysis and writing-up of the manuscript. TMY participated in study design, participated in data collection, and write-up. All authors read and approved the final manuscript.

## Pre-publication history

The pre-publication history for this paper can be accessed here:

http://www.biomedcentral.com/1472-6874/14/50/prepub
